# Boquila: NGS read simulator to eliminate read nucleotide bias in sequence analysis

**DOI:** 10.55730/1300-0152.2650

**Published:** 2023-02-21

**Authors:** Ümit AKKÖSE, Ogün ADEBALİ

**Affiliations:** 1 Faculty of Engineering and Natural Sciences, Sabancı University, İstanbul, Turkey; 2 TÜBİTAK Research Institute for Fundamental Sciences, Gebze, Turkey

**Keywords:** NGS, sequence read simulation, nucleotide content

## Abstract

Sequence content is heterogeneous throughout genomes. Therefore, genome-wide next-generation sequencing (NGS) reads biased towards specific nucleotide profiles are affected by the genome-wide heterogeneous nucleotide distribution. Boquila generates sequences that mimic the nucleotide profile of true reads, which can be used to correct the nucleotide-based bias of genome-wide distribution of NGS reads. Boquila can be configured to generate reads from only specified regions of the reference genome. It also allows the use of input DNA sequencing to correct the bias due to the copy number variations in the genome. Boquila uses standard file formats for input and output data, and it can be easily integrated into any workflow for high-throughput sequencing applications.

## 1. Introduction

Simulating genomic data for benchmarking bioinformatics programs has become increasingly popular, particularly for read alignment, genome assembly, and variant and RNA-seq analysis (Mangul et al., 2019). Using such an approach allows for systematic performance assessment even in the absence of gold-standard data. Most currently available simulation tools are heavily geared towards benchmarking; they concentrate on generating reads produced by a specific sequencing experiment by modeling the characteristics of reads produced by sequencing machinery. Consequently, the correction metrics are primarily associated with artificial errors commonly introduced by these specific sequencing protocols.

Although most of the tools use some profile for simulating, these profiles are used for simulating characteristics of sequencing protocols rather than biological experiments. Nucleotide content profile (nucleotide profile) is the proportion of each nucleotide out of the implied four total nucleotides on a position basis. No simulation tool utilizes nucleotide content profile to our knowledge. SomatoSim (Hawari et al., 2021), VarSim (Mu et al., 2015), SimuSCoP (Yu et al., 2020), and many other tools (Pattnaik et al., 2014; Qin et al., 2015; Ivakhno et al., 2017; Xia et al., 2017; Yuan et al., 2017) were specifically designed to simulate genomic variation. ART (Huang et al., 2012) and SInC (Pattnaik et al., 2014) generate profiles based on error models and quality score distribution extracted from empirical data. pIRS (Hu et al., 2012) and Mitty (ozemsbg, 2018) generate quality profiles based on mapped reads and empirical data. NanoSim (Yang et al., 2017), a nanopore sequence simulator, also uses error profiles and length distributions. Gargammel, an ancient DNA sequencing simulator, uses sequencing errors (misinterpreted signals by sequencers or incorporation of wrong nucleotides) and quality profiles can model base compositions. It can mimic UV damage by adding deamination. However, it is specifically designed for simulation of ancient DNA sequences (Renaud et al., 2017). BEAR (Johnson et al., 2014), focused on metagenomics, generates error, quality, and abundance profiles. The method developed by Wang et al. (Wang et al., 2017) can be used for detecting and correcting position-specific biases in short read data. It corrects position-specific imbalances across reads by calculating read specific weights based on aligned reads. It has a different purpose and cannot be used generating simulated datasets. However, the nucleotide content of the reads could be biased for several reasons. First, if sequence library preparation involves immunoprecipitation, antibodies might be biased towards pulling down specific nucleotide profiles. Moreover, ligation efficiencies could be different across varying nucleotides on both 5′ and 3′ ends, which would result in some nucleotide enrichment at the read ends. Furthermore, the nature of sequencing technology might supposedly yield a particular biased nucleotide profile. For instance, sequencing methods yielding the maps of UV damage naturally result in dipyrimidine-enriched reads (Hu et al., 2016; Mao et al., 2016). Additionally, the GC content, percentage of nitrogenous bases in a DNA or RNA that are either guanine or cytosine, of the reads might vary depending on the sequencing platform (Ross et al., 2013). Finally, the polymerase chain reaction (PCR) step might introduce another nucleotide bias due to differential efficiencies of universal primers towards specific nucleotides (Polz and Cavanaugh, 1998). Considering such factors that could affect the genomic distribution of the reads, there is a clear need for a sequencing read simulation tool that utilizes nucleotide profile although simulated reads can also be used to eliminate nucleotide content bias in experiments whose results are affected by nucleotide content. Simulation tools mentioned above can account for error and quality profiles of sequencing platforms and GC content biases. However, most of them were designed to simulate reads based on sequencing instruments. There was no other option if aimed to generate a simulated dataset that mimics the nucleotide content of input reads.

Here we present boquila, a nucleotide content-based NGS read simulator that can produce simulated reads with a similar nucleotide profile to input reads. They can be used to normalize the nucleotide content bias in actual reads by getting fold change between simulated reads and actual reads.

Genomic regions with higher copy numbers have a greater chance of being pulled down during library preparation, whereas those with lower copy numbers are harder to detect.

Boquila can also use data from input sequencing as input while generating simulated reads. With this approach, we can also use generated simulated reads to normalize the impacts of copy number variations (CNV).

## 2. Features and methods

Boquila was specifically developed to generate synthetic reads while mimicking the nucleotide content of input reads. It operates with FASTA or FASTQ files as an input and generates reads according to the nucleotide content of the input. The number of generated reads and their length distribution will equal the input reads, but if there are ambiguous nucleotides (N) in the input reads, those reads will be discarded and will not be used for simulation. The nucleotide profile can be calculated based on user-defined k-mer length or single nucleotides. Boquila can use the entire genome or predefined genomic intervals while randomly selecting reads from the reference genome, thus providing fine-grained control over the regions where simulated reads are generated. Alternative to the reference genome, input sequencing reads can also be used, if a user has raw genome sequence data as a control retrieved from the same experimental setup (cell type, conditions, etc). In this case, reads are randomly generated from input sequencing reads. When generating simulated reads, the nucleotide profile obtained from input reads is adjusted dynamically based on the nucleotide profile obtained so far from simulated reads. In this manner, simulated reads can be further conformed to input reads.

Simulated reads are exported in FASTA or FASTQ format based on the input read format.

Simulated reads can also be exported in BED format. Quality scores are copied over from input reads if input reads are present in FASTQ format to keep the mappability of simulated reads the same as input reads. As an alternative option, user-selected quality values can be applied to each generated read. FASTA and FASTQ are standard formats for high-throughput sequencing reads, making boquila easier to integrate into existing workflows. Additionally, obtaining output in BED format can help bypass alignment, which is one of the most time- and resource-intensive steps in any NGS workflow.

### 2.1. Read simulation

**Figure 1:**
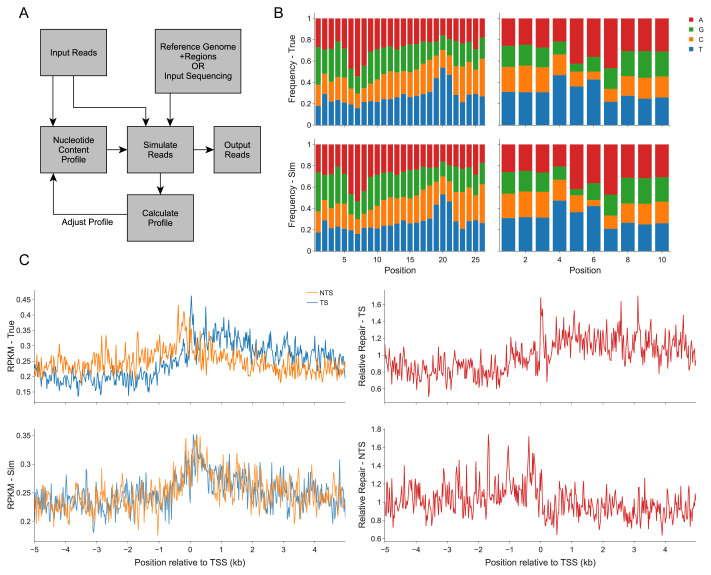
(A) Overall workflow of boquila. (B) Nucleotide frequency of simulated and true reads for XR-seq (left) and damage-seq (right). XR-seq reads are enriched with thymines at 19th–21st positions of the reads, whereas centered damage-seq reads are enriched with pyrimidines at 5th–6th positions. (C) UV-induced repair (XR-seq) profiles around transcription start sites (TSS). Due to transcription-coupled repair (TCR), transcribed strand (TS) is expected to have higher repair signals relative to the nontranscribed strand (NTS). Whether sequence bias affect the TCR profile around TSS is investigated with the simulated reads (bottom). The profiles showing observed/expected (due to sequence context) ratios (on the right) indicate the TS (top) and NTS (bottom) TCR profiles. RPKM: reads per kilobase per million mapped reads.

Boquila first calculates the nucleotide profile (NP) of input reads (Figure 1A). For each record in input, we uniformly sample 50 (adjustable: can be decreased for faster read generation, or can be increased to make the profile of simulated reads more similar to the input reads if necessary) records from the reference genome. Next, score for each read is obtained where each nucleotide in the record was scored based on the frequency of that nucleotide to be in that position in the input reads using NP of input reads. The NP used in read generation is adjusted after every 10% of reads are generated by adjusting it using the difference between NP of input reads and NP of reads simulated so far. Half of the difference between NP of simulated reads until that point and NP of input reads (observed NP) is subtracted from NP used for simulation process to converge to the observed NP. Convergence is processed after each decile of the simulated reads which were randomly selected from the reference genome or input DNA sequencing. Therefore, the order of input reads does not affect this convergence process.

### 2.2. Performance

To test boquila’s speed, we generated reads for *Escherichia coli* excision repair sequencing (XR-seq) data (Adebali et al., 2017). The test was performed on a compute cluster with Intel Xeon Gold 6140 CPU @ 2.30GHz, running Linux operating system. The test took <14 min (Table 1), with Fasta input format being slightly faster than Fastq. We also generated some fixed length reads (10bp and 20bp), runtime increased linearly with respect to number of simulated reads. And doubling read length increased runtime 60%.

## 3. Results

We used boquila to generate simulated reads for the data sets of two published studies: XR-seq data from (Adar et al., 2016) and damage-seq data from (Hu et al., 2017). Simulated reads have the similar nucleotide content as true reads (Figure 1B) (XR-seq Hotelling’s T-square: 0.25, p-value: 0.99, damage-seq Hotelling’s T-square: 5.2, p-value: 0.4), but they are randomly generated from the genome. Both XR-seq and damage-seq reads have a certain nucleotide bias due to the UV-induced damage site. The simulated reads generated by boquila can be used to normalize the true repair signal and the true damage signal obtained from XR-seq and damage-seq, respectively by getting fold change between simulated signal and true signal (Figure 1C). Using this method, we can eliminate the potential bias of the nucleotide content in subsequent analyses.

## 4. Discussion

Nucleotide content of the reads can be biased for several reasons, including antibody bias immunoprecipitation, ligation efficiency of nucleotides, and the nature of used sequencing technology. These biases should be normalized before any downstream analysis, but currently, no tools are available for normalizing nucleotide content bias. In this study, we propose a novel sequencing simulator utilizing nucleotide content. Some simulators can account for bias produced by sequencing machinery and GC content. Furthermore, some focused on introducing genomic variation into simulated reads (Table 2). However, no simulation tool can yield random reads reflecting the nucleotide content profile of the input reads. Alternative to the reference genome, boquila can also use input sequencing data during simulation to account for CNV bias, which most other simulation tools neglect. With the development of NGS technologies for each specific research question, specialized correction tools must be used to remove technical artifacts of the methodology. In that respect, boquila fills an essential gap in NGS analysis.

The simulation of quality scores was not included as a feature. However, specific quality scores can be applied to simulated reads or transferred from input reads if necessary. The simulation of sequencing errors or genetic variations is not supported by boquila either. Boquila’s main goal was to create simulated datasets with nucleotide content profiles similar to input reads. Hence, these features were left out, but in order to incorporate such elements in simulated reads subsequently, other simulation tools could be used.

## Figures and Tables

**Table 1:** Boquila simulation speed.

Input format	Read length	Number of reads	Runtime (s)	Speed (no. of reads/s)
Fasta	Varied (17–31 bp)	15,279,119	819	18,655
Fastq	Varied (17–31 bp)	15,279,119	838	18,232
Fasta	10 bp	15,279,119	601	25,422
Fasta	10 bp	7,639,559	295	25,896
Fasta	20 bp	15,279,119	1049	14,565
Fasta	20 bp	7,639,559	511	14,950

**Table 2:** Brief summary of existing simulation tools. (SE: single end, PE: paired end, FA: fasta, FQ: fastq, SNV: single nucleotide variation, CNV: copy number variation, SV: structural variation.)

Simulator	Output layout	Output format	GC bias	CNV bias	Nucleotide content	Genomic variation	Simulated profile
MetaSim (Richter et al., 2008)	SE	FA					Empirical error, genome abundance profiles
Mason (Holtgrewe, 2010)	SE, PE	FA, FQ					Empirical error and quality profiles
BEERS (Grant et al., 2011)	PE	FA					Intron signal profile, error profile
ART (Huang et al., 2012)	SE, PE	FQ, SAM					Empirical length, error, quality profiles
GemSIM (McElroy et al., 2012)	SE, PE	FQ, SAM	✓			✓	Empirical quality and error profiles
Grinder (Angly et al., 2012)	SE, PE	FA, FQ		✓			Position dependent error profile
pIRS (Hu et al., 2012)	PE	FQ	✓			✓	Empirical base-calling, gc%- depth profiles
Wessim (Kim et al., 2013)	SE, PE	FQ, SAM	✓				fragment generation models
NeSSM (Jia et al., 2013)	SE, PE	FQ	✓				Error and coverage profiles
xs (Pratas et al., 2014)	SE, PE	FQ					Read-length and quality profiles
SInC (Pattnaik et al., 2014)	SE, PE	FQ				✓	Quality and error profiles
CuReSim (Caboche et al., 2014)	SE	FQ	✓			✓	Error profile
FASTQSim (Shcherbina, 2014)	SE	FQ					Read-length, quality and error profiles
BEAR (Johnson et al., 2014)	SE, PE	FQ					Empirical length, error, quality profiles
VarSim (Mu et al., 2015)		FQ				✓	Mutation profile
SCNVSim (Qin et al., 2015)	SE, PE	FA, VCF				SV, CNV	-
IntSim (Yuan et al., 2017)	SE, PE	FQ	✓			✓	Read-length, quality and error profiles
tHapMix (Ivakhno et al., 2017)		BAM				✓	Error profile
gargammel (Renaud et al., 2017)	SE, PE	FQ	✓				Damage, fragmentation, contamination profiles
NEAT (Stephens et al., 2016)	SE, PE	FQ	✓			✓	Read-length, quality and error profiles
NanoSim (Yang et al., 2017)	SE	FQ					Error profile, length distribution profile
Pysim-sv (Xia et al., 2017)	SE, PE	FQ	✓			✓	-
Xome-Blender (Semeraro et al., 2018)	PE	BAM				✓	-
Mitty (ozemsbg, 2018)	SE, PE	FQ, VCF	✓			✓	Empirical quality and length profiles
PaSS (Zhang et al., 2019)	SE, PE	FQ					Read-length and error profiles
SimuSCoP (Yu et al., 2020)	SE, PE	FQ	✓			✓	Quality and error profiles
SomatoSim (Hawari et al., 2021)	PE	SAM, BAM				SNV	Error profile
Boquila	SE	FA, FQ, BED	✓	✓	✓		Nucleotide content profile

## Data Availability

Boquila is written in Rust and freely available at https://github.com/CompGenomeLab/boquila.
